# How and When Health-Promoting Leadership Facilitates Employee Health Status: The Critical Role of Healthy Climate and Work Unit Structure

**DOI:** 10.3389/fpsyg.2021.727887

**Published:** 2021-12-24

**Authors:** Shuang Liu, Zhimin Song, Jincen Xiao, Peimin Chen

**Affiliations:** ^1^School of Business Administration, Hebei University of Economics and Business, Shijiazhuang, China; ^2^School of Business Administration, Southwestern University of Finance and Economics, Chengdu, China; ^3^School of Management, Xihua University, Chengdu, China; ^4^School of Hotel Administration, Shanghai Business School, Shanghai, China

**Keywords:** health-promoting leadership, social information processing theory, healthy climate, work unit structure, employee health status

## Abstract

Health-promoting leadership has attracted a lot of attention from scholars in recent years. However, majority studies focused on theoretical arguments rather than empirical examination. Not only that, extant research often theorizes health-promoting leadership as a combination of a series of direct and explicit health-related behaviors, neglecting the potential social information it may convey to employees. Based on social information processing theory, this study empirically examines *how* and *when* health-promoting leadership can facilitate employees’ health status. Using a time-lagged data of 370 employees (i.e., matched to 51 leaders), we found that health-promoting leadership has a significant and positive influence on employees’ health status, and healthy climate acts as a linking pin. In addition, work unit structure moderates the relationship between health-promoting leadership and healthy climate. Specifically, compared with mechanic work unite structure, employees rely more on social information conveyed by health-promoting leadership when working at an organic work unite structure. This study not only extends current knowledge about the effect of health-promoting leadership, but also provides useful guidance for practitioners.

## Introduction

Nowadays, doing a job is not just to make a living, but to get joy and meaning across the lifespan. Indeed, a job provides employees with a lot of meaningful things such as financial resources, psychological support, and interpersonal interaction. However, a lot of issues in the workplace are detrimental to employee health such as increasingly urgent task and long working hours. Given employee health is important for organizational sustainable development, scholars have long been interested in exploring how to promote employee health (Montano et al., 2017). Extant research has verified leaders as a crucial factor in facilitating employee health because of their ubiquitous and intensive influence on employees (e.g., Gurt et al., 2011; Gregory and Osmonbekov, 2019). As the most direct leadership approach concerning employee health, health-promoting leadership (also termed as health-oriented leadership, Krick et al., 2021; health-specific leadership, Gurt et al., 2011) thus has received abundant attention from scholars in recent years (e.g., Akerjordet et al., 2018; Turgut et al., 2020; Yao et al., 2021).

Health-promoting leadership not only tries to take health-related responsibility for employees (Gurt et al., 2011), but also aims to create a culture for health-promoting workplace and values that inspire employees to participate in such a development (Eriksson et al., 2011). In general, as suggested by Jiménez et al. (2017b), health-promoting leadership can promote the health of employees by facilitating some key factors such as health awareness, value-fit, fairness, community, reward, control, and low workload. Empirical evidence has demonstrated that health-promoting leadership is positively to employee engagement (Liu et al., 2021), well-being (Kaluza et al., 2021), while negatively related to employee burnout (Jiménez et al., 2017a). However, existing research theorizes health-promoting leadership as a combination of a series of direct and explicit health-related behaviors, neglecting the potential social information it may convey to employees. Not only that, since the regime of “996” (i.e., “working from 9 am to 9 pm, 6 days a week without extra pay for those extra hours,” Wang, 2020, p. 4331) has been adopted by some employers in the past few years, it is necessary to investigate how to promote employee health in current Chinese organizations (Liu et al., 2021).

This study aims to explore how and when health-promoting leadership influence employee health status. Since health-promoting leadership can promote employee health by directly showing them health awareness or by indirectly cultivating health work condition (Franke et al., 2014; Jiménez et al., 2017b), it could be considered as a crucial source of social information (Schriesheim and Liu, 2018; Lu et al., 2019). This study adopts social information processing theory as theoretical foundation (Salancik and Pfeffer, 1978). Social information processing theory contends that individuals use information from their work environments to interpret events and decide how to behave (Salancik and Pfeffer, 1978). Notably, although the notion that leader behaviors are powerful social information has been verified in several types of leaderships such as servant leadership (Lu et al., 2019), shared leadership (Ali et al., 2021), and authentic leadership (Schriesheim and Liu, 2018), health-promoting leadership has not yet been empirically tested. Hence, investigating the effect of health-promoting leadership from social information processing theory is a crucial step moving forward research on health-promoting leadership.

Furthermore, previous research has shown that health-promoting leadership is positively related to employee health (Kaluza et al., 2021; Yao et al., 2021). However, conceptualizing its underlying effect as solely direct is insufficient. That it, research on health-promoting leadership usually implicitly assumes mediating factors without explicitly testing them. We thus consider healthy climate as a key linking pin. Healthy climate refers to employees’ shared perception about organization’s expectations, recognition and encouragement for health practices, procedures, and behaviors, which generally includes organizational health norms, values and practices, health promotion programs and environmental conditions (Kerr et al., 2019). In addition, since the influence of leadership is contingent upon work conditions, this study also aims to examine the boundary condition of the effect of health-promoting leadership. We consider work unit structure as a situational moderating variable. Work unit structure reflects the characteristics of an organization’s internal environment and affects information communication and behavior changes among members of an organization (Nielsen et al., 2017), which can be divided into mechanistic and organic type (Aryee et al., 2008). This framing is also consistent with social information processing theory as it suggest that to what extent individuals rely on social information for guidance depends on the clarity of their working environment (Salancik and Pfeffer, 1978).

**FIGURE 1:**
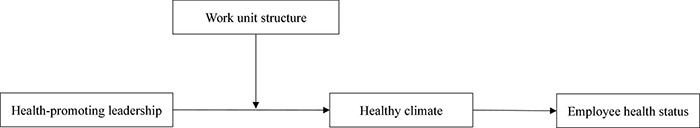
The proposed model of current research.

In sum, this study develops a theoretical model linking health-promoting leadership to employee health status with healthy climate as mediating mechanism and work unit structure as boundary condition. To test our theoretical model, we collect paired date of 51 leaders and 370 employees in Chinese organizations. This study makes several contributions to the existing literature. First, we examined the effect of health-promoting leadership on employee health status from the perspective of social information processing theory. To our best knowledge, the consequences of health-promoting leadership has not been explored from this perspective. Second, we uncovered the mediating mechanism underlying the relationship of health-promoting leadership and employee health status. That is, we illustrated that one reason the health-promoting leadership promotes employee health status is the strengthened healthy climate. Third, we verified a work unit level factor (i.e., work unit structure) as boundary condition of the downward effect of health-promoting leadership. Figure 1 presents our proposed research model.

## Theory and Hypotheses

### Social Information Processing Theory

According to social information processing theory, individuals observe and process social information from their work environments to interpret events and decide how to behave based on their interpretations (Salancik and Pfeffer, 1978). Notably, leaders are powerful sources of this social information, given their power and authority to control and influence employees’ resources (Lu et al., 2019). Indeed, social information processing theory has often been adopted to understand the effect of certain leaderships. For example, Boekhorst (2015) argued that authentic leadership can transmit social information about the importance of inclusion into the work environment through inclusive leader role modeling. In a similar vein, ethical leadership shapes employee creative performance because such leadership drives employee to feel respected and trusted to identify organizational problems, search for solutions, and come out with novel and useful ideas (Wadei et al., 2021).

Health-promoting leadership not only focuses on direct health-related behaviors such as purchasing ergonomic office supplies, but also pays attention to some obscure behaviors such as emphasizing health awareness (Jiménez et al., 2017b). As a result, employees must go through deliberated cognitive processes to understand the social information delivered by leaders. By interpreting the social information of health-promoting leadership behavior, employees are more willing to accept the meaning of health and produce healthy work behavior in the workplace. From this point of view, health-promoting leadership can improve the health status of employees by establishing a healthy image, capturing health risks, and taking health measures. Furthermore, through the exertion and guidance of health-promoting leadership, the members of the organization continuously interpret the health information in the organizational environment during the interaction with the organization, and gradually form a state of consensus in the aspects of heath concepts, behaviors, and expectations. In sum, drawing on social information processing theory (Salancik and Pfeffer, 1978), this study aims to develop a social influence model to explore the effect of health-promoting leadership.

### Health-Promoting Leadership and Healthy Climate

Leadership is generally considered to be a key factor in influencing workplace climate (Wallace et al., 2011). In fact, leaders are the policy makers and implementers in the organization. They guide the formation of organizational climate and influence the characteristics of organizational climate by formulating organizational rules and regulations or influencing the implementation process of specific policies and measures (Franke et al., 2014). Health-promoting leadership can promote employees to accept the concept of health and subsequently participate in health promotion activities (Yao et al., 2021). Not only that, health-promoting leadership is expected to enhance employees’ recognition of health values by formulating workplace health protection measures, implementing health promotion projects, and establishing reward and punishment system (Nelson et al., 2015).

According to social information processing theory, leader behavior is an important source of social information for employees (Salancik and Pfeffer, 1978; Boekhorst, 2015). Health-promoting leaders actively promotes their own health concept and vision in the workplace, and establishes a healthy communication mechanism with employees. By doing so, they are expected to help employees form a consistent health cognition. Essentially, workplace climate is a signal source that provides employees with organizational expectations, values, and codes of conduct (Schneider et al., 2013; Zadow et al., 2017). Through observing the surrounding environment and judging the past behaviors, employees can acquire a series of information such as what the organization is supporting. Healthy climate is an indicator of the organization’s health values and health behavior standards, which cultivates employees to realize that their organizations encourage them to maintain and improve their own health status. The communication between employees will further confirm and strengthen their perception of the healthy climate of the organization. As a result, the healthy climate is constantly deepened and improved, which ultimately forms a clearly unified cognition in the organization. Therefore, we hypothesize the following:

Hypothesis 1: Health-promoting leadership is positively related to healthy climate.

### Healthy Climate and Employee Health Status

Organizational climate is an important part of work environment. The interaction of organizational members in the workplace will form the expression of individual will, expectation and preference (Schneider, 1987). This expression gradually tends to be consistent in the process of mutual communication and sharing, and this consistent gathering rises to the structural characteristics of the organization or team level, and has an impact on the entire organization or team (Schneider et al., 2013). Organizational climate can form a source and distribution center of information through the interaction among members. This interactive process includes not only the communication and sharing between colleagues, but also the communication and coordination between the leaders and employees. Accordingly, organizational members’ thinking and understanding of the working environment will form the principle of sharing and communicating among members, which subsequently affects their attitude and behavior (Boekhorst, 2015).

Organizations are increasingly aware of their important role in helping employees achieve healthy work (Goetzel and Ozminkowski, 2008). First, through the formulation of health-related policies, measures, and procedures, the organization creates a healthy environment and climate for employees, which affects the change of their health behavior, and promotes the improvement of individual health status. Second, a healthy climate can reduce the stress of employees. As suggested by Kerr et al. (2019), healthy climate includes the element of favorable organizational climate and benign relationship climate. Cultivating a healthy climate can reduce work resistance or promote work motivation. Ribisl and Reischl’s (1993) research shows that when employees perceive their organizations pay greater attention to health problems, they are likely to feel lower work pressure and higher job satisfaction. More recently, Schwatka et al. (2020) found that organizational healthy climate can facilitate employee health-related behaviors because such climate enhances their intrinsic, identified, and external motives. In sum, healthy climate represents popularity of health concept among employees and successful implementation of health-related policies, which is ultimately beneficial to employee health status.

Furthermore, healthy climate can help employees develop good health habits. As suggested by Franke et al. (2014), healthy climate can affect employees’ attitude and help them form healthy living habits. Organization plays an important part of employees’ health because it can affect employees’ attitudes and behaviors. Many employees can choose the time of 1 day to exercise, which stimulates their energy at work (Neal et al., 2000). In addition, receiving support from supervisor and colleague have a negative impact on physical illness, and a positive impact on job satisfaction (Yang et al., 2019). Therefore, perceived support for health-related behaviors helps employees develop specific health habits. For example, if nutrition and fitness guidelines are added to the health regulations, employees are more likely to develop healthy eating and exercise habits., which enhances this health status (Crane et al., 2019). Therefore, we hypothesize the following:

Hypothesis 2: Healthy climate is positively related to health status.

### Healthy Climate as a Mediator

Leaders are the designer and facilitator of organizational climate (Skakon et al., 2010). By influencing the formulation and implementation of organizational rules and regulations, leaders can impact the relationship among members at workplace. In addition, leaders can manipulate or control other important factors that influence the formation of the climate through their own power and influence (Wadei et al., 2021). These factors have an impact on employees’ attitude choice and behavior style. In the specific operation process, health-promoting leadership needs to take a series of specific management approaches (e.g., designing appropriate structure) to transmit their own health concept, team health purpose, organization health support and other information to the organization (Yao et al., 2021). Employees can communicate with each other through understanding information and behavior exploration to continuously confirm the attitude of the organization to its health status. When employees’ understanding of organizational information tends to be consistent, a healthy climate is expected to be formed. The employees who are exposed to the healthy climate have a clear health concept, good health awareness and positive health behavior.

Healthy climate includes several ways to improve employee health status such as meeting the needs of employees within a reasonable range, strengthening the control of employees on their work, and providing knowledge sharing required by work (Neal et al., 2000). As suggested by social information processing theory, organization members will process information source provided by the organization before making judgment and taking actions (Salancik and Pfeffer, 1978). As an important clue provided by the environment, the health concept, health awareness, and health behavior of leaders convey the message that paying attention to health is beneficial to team members (Pipe et al., 2012). Through the communication with leaders, employees are likely to form some shared perceptions. For example, it is very important to achieve mental and physical health and such behaviors will be encouraged and supported by their working teams. As the healthy climate gradually forms, employees’ health concept, health awareness and health behavior are likely to be influenced. In view of this, this study suggests that healthy climate can play a mediating role between health-promoting leadership and health status. Therefore, we hypothesize the following:

Hypothesis 3: Healthy climate mediates the positive relationship between health-promoting leadership and employee health status.

### The Moderating Role of Work Unit Structure

Work unit structure is considered as the relationship between people in a work unit (Aryee et al., 2008), which involves many context factors such as interpersonal relationship, decision-making pattern, and communication approach. According to the social information processing theory, social environment will affect the individuals’ attitudes and needs in organizations (Salancik and Pfeffer, 1978). When the working environment changes, individuals tend to adjust their attitudes, behaviors, and beliefs to adapt to meet their needs of adapting to the social environment. During the self-regulation process, environment becomes an important information source. By interacting with environment, various information in the environment constantly corrects and shapes individuals’ attitudes and needs. In sum, work unit structure is expected to be a crucial boundary condition of the effect of health-promoting leadership because it determines how to do things here (Schneider, 1987).

Furthermore, work unit structure describes the “Sum total of the ways in which labor is divided into distinct tasks and coordination is achieved among them” (Mintzberg, 1979, p. 2), which ranges from mechanistic to organic type (Aryee et al., 2008). More frankly speaking, work unite structure determines to what extent work activities are structured (Dragoni and Kuenzi, 2012). If one’s work activities are highly structured, his or her roles will be clearly defined by task specialization, reporting object, and standard routines. Not only that, structured work activities also represent concentration of authority, which means decision-making authority is concentrated at the relatively top of the hierarchy (Dragoni and Kuenzi, 2012). In this case, employees cannot make decisions freely, and their decisions are often be neglected by leaders or organizations. Moreover, work unit structure also includes line control of the workflow (Dragoni and Kuenzi, 2012). This feature describes the extent to which one can control the workflow. Based on above arguments, we argue that work unit structure determines whether work activities are uncertain or ambiguous.

Specifically, we propose that health-promoting leadership is more positively related to healthy climate when the work unit structure is organic. In the organic work unit structure, interpersonal interactions are close and accessible due to reliable information network and face-to-face communication (Aryee et al., 2008). Members in such work unit are more likely to obtain information through their free interaction. Organic structures are more fluid structures that are characterized by a lack of formally defined tasks and decentralized decision making, which means they are uncertain and ambiguous (Yang, 2017). As suggested by social information processing theory (Salancik and Pfeffer, 1978), when situations are uncertain, ambiguous, and complex, individuals have a stronger desire to rely on social cues to form their work attitudes and behaviors. Therefore, we expect a stronger relationship between health-promoting leadership and healthy climate under the organic work unit structure. In contrast, mechanistic structures are highly formalized and centralized (Aryee et al., 2008). Due to its rigorous and bureaucratic characteristics, leaders’ health-related behaviors are difficult to influence employees. Not only that, employees have a clear working guidance in mechanistic work unit structure, which means they do not need to rely on social information to guide their behaviors (Salancik and Pfeffer, 1978). Therefore, we hypothesize the following:

Hypothesis 4: Work unit structure moderates the relationship between health-promoting leadership and healthy climate, such that the positive relationship between health-promoting leadership and healthy climate will be stronger in the organic work unit structure mechanistic structure but weaker in the mechanistic work unit structure.

## Materials and Methods

### Samples and Procedures

To test our theoretical model, we conducted a time-lagged survey study in China. The time-lagged survey design was used to minimize common method variance (Podsakoff et al., 2003). We reached out to three organizations after obtaining consent and receiving ethical approval from its top managers. We introduced our research purpose and procedures to the participants. We informed them that participation was voluntary and their responses would be kept confidential and only used for research purposes. Participants received a ¥20 cash coupon (about $3) for their kind participations. In order to rule out the interpersonal interferers in the filling process, we tried to separate employee from leaders in space.

More specifically, in the time 1 survey, participants rated on their perception of health-promoting leadership and work unit structure, and filled their demographic information. In this round, we collected 474 questionnaires. In the time 2 survey, we distributed questionnaires to employees who had joined the time 1 survey, and asked them to assess healthy climate and health status. By matching employees to their respective workgroup supervisors *via* a unique identification code, our final sample consisted of 370 employees nested in 51 teams. Among the sampled employees, 46.13% were male, 60.125% were less than 35 years old, and 72.861% have more than 3 years of organizational tenure.

### Measures

Three graduate students majoring in human resource management were invited to conduct a parallel, double-blind “translation back translation” procedure (Brislin, 1980). All questionnaires were measured by Likert’s 5-point scoring system, from “1” = strongly disagree to “5” = strongly agree. The Appendix 1 shows the items for each construct.

#### Health-Promoting Leadership

Health-promoting leadership was rated on the 3-item scale developed by Franke et al. (2014). A sample item was “If I feel unwell, my boss will take immediate action.” The Cronbach’s alpha was 0.814.

#### Healthy Climate

The 5-itm scale developed by Basen-Engquist et al. (1998) was used to measure healthy climate. The original scale consisted of safety climate and healthy climate sub-scales. In this study, the sub scale of healthy climate was employed. A sample item was “In my workplace, sometimes we talk to each other about how to improve health and prevent diseases.” The Cronbach’s alpha was 0.773.

#### Work Unit Structure

We used the 7-item scale developed by Aryee et al. (2008) to measure work unit structure. Each employee was asked to rate the extent to he or she agree with the statements. A sample item was “Leaders can operate in any way from very formal to very informal.” The Cronbach’s alpha was 0.801.

#### Employee Health Status

Employee health status was measured on the 12-item general health questionnaire (GHQ-12) developed by Goldberg and Williams (1988). A sample item was “Thinking of self as worthless.” The Cronbach’s alpha was 0.858.

#### Control Variables

In this study, we controlled the factors that may affect the health status of employees, including the gender, age, and organizational tenure. Specifically, we control for gender because there are significant differences between men’s and women’s health attitudes. Compared with younger employees, older employees are more likely to pay more attention to their health. In addition, the length of organizational tenure will affect the sensitivity of employees to the health elements of organizational culture, workflow, and information communication (Jiménez et al., 2017a). Therefore, gender, age and organizational tenure were taken as control variables in this study. According to Bernerth and Aguinis’s (2016) recommendations, we also performed all the analysis without control variables and found that results were remain supported.

### Analytic Technique

In view of the nested structure of data (i.e., multiple employees report to one supervisor), we used multilevel structural equation modeling in Mplus 7.0 (Muthén and Muthén, 2017). Before the analysis, we made a centralized treatment on the average of the employees’ gender, age, and organizational tenure. Then, the group mean centralization was carried out on health-promoting leadership, healthy climate, and work unit structure. The Monte Carlo method recommended by Preacher and Selig (2012) was used to estimate the confidence interval (*CI*s) of the hypothesized mediation. To test for moderation effect, we evaluated the effect at “high” (one standard deviation above the mean) and “low” (one standard deviation below the mean) values of the moderator (i.e., work unit structure).

## Data Analysis and Results

### Confirmatory Factor Analysis

We conducted a multilevel confirmatory factor analysis to test the discriminative validity of the focal variables in our model. Given that the ratio of the sample size and the total number of items impairs overall model fit, scholars suggested that using item parcels could reduce the number of parameters and mitigate the impairment (Little et al., 2013). As recommended by Kishton and Widaman (1994), we parceled variables using the domain-representative approach, which means parcels were created by joining items from each dimension into item sets. The results showed that the hypothesized four-factor model provided a good fit to the data: *x*^2^ = 202.06; *df* = 129; *SRMR*_*within*_ = 0.02; *SRMR*_*between*_ = 0.19; *RMSEA* = 0.03, *CFI* = 0.96; *TLI* = 0.95, and the fit indices are superior to any other alternative models (see Table 1). Therefore, construct distinctiveness of the main variables is established.

**TABLE 1:** Comparison of measurement models.

Models	χ^2^	*df*	Δχ^2^ (*df*)	RMSEA	χ^2^/*df*	TLI	CFI
Hypothesized 4-factor model (HL, HC, WUS, HS)	202.06	129		0.03	1.57	0.95	0.96
Alternative 3-factor model (HL + HC, WUS, HS)	447.71	132	245.65*** (3)	0.07	1.74	0.80	0.83
Alternative 3-factor model (HL, HC + WUS, HS)	445.13	132	243.07*** (3)	0.07	3.37	0.81	0.82
Alternative 2-factor model (HL + HC + WUS, HS)	908.81	134	706.75*** (5)	0.11	6.78	0.51	0.58
Alternative 1-factor model (HL + HC + WUS + HS)	1,315.54	135	1,113.48*** (6)	0.13	9.74	0.26	0.35

*N = 370 employees; all models were compared with the hypothesized four-factor model.*

*HL, health-promoting leadership; HC, healthy climate; WUS, work unit structure; HS, employee health status; RMSEA, root mean square error of approximation; CFI, comparative fit index; TLI, Tucker–Lewis index.*

***p < 0.001.

### Hypothesis Tests

Table 2 shows the means, standard deviations, intercorrelations, and internal consistencies of variables in this study. Results indicate a rational association between theory and statistics. For example, health-promoting leadership was positively related to healthy climate (*r* = 0.213, *p* < 0.001), and healthy climate was positively related to employee health status (*r* = 0.191, *p* < 0.001).

**TABLE 2:** Means, standard deviations, inter-correlations, and internal consistencies of studied variables.

Variables	Mean	SD	1	2	3	4	5	6	7
1. Employee gender	1.55	0.50	–						
2. Employee age	2.24	0.82	−0.173***	–					
3. Employee organizational tenure	3.27	1.46	−0.106*	0.670***	–				
4. Health-promoting leadership	3.61	0.85	–0.013	0.051	–0.058	(0.814)			
5. Healthy climate	3.56	0.17	–0.057	0.144**	0.101*	0.213***	(0.773)		
6. Work unit structure	4.07	0.45	–0.038	0.048	–0.039	0.232***	0.104*	(0.801)	
7. Employee health status	2.40	0.65	0.039	0.082	0.028	0.179***	0.191***	0.077	(0.858)

*N = 370 employees.*

*Gender: 1 = male; 2 = female.*

*Age: 1 = 24 years old or below; 2 = 25–35 years old; 3 = 35–45 years old; 4 = 46 years old or above.*

*Organizational tenure: 1 = 1 year or below; 2 = 1–2 years; 3 = 3–5 years; 4 = 6–10 years; 5 = 10 years or below.*

*Cronbach’s alphas are reported in the parentheses on the diagonal.*

*** p < 0.001, **p < 0.01, and *p < 0.05.

Table 3 presents the results of our hypothesis testing. Hypothesis 1 proposes that health-promoting leadership is positively related to healthy climate. Consistent with this hypothesis, the results showed that the relationship between health-promoting leadership and healthy climate was significant (*b* = 0.146, *SE* = 0.012, *p* < 0.01). Hypothesis 2 predicts that healthy climate is positively related to employee health status. As shown in Table 3, healthy climate is significantly and positively related to employee health status (*b* = 0.449, *SE* = 0.131, *p* < 0.01). Accordingly, Hypothesis 3 suggests that the healthy climate mediates the relationship of health-promoting leadership and employee health status. The results showed that the 95% CI for the indirect effect did not include zero [*b* = 0.09, 95% CI = (0.028, 0.106)], supporting Hypothesis 3.

**TABLE 3:** Hierarchical linear modeling results.

Variables	Mediating effect		Moderating effect
	Healthy climate	Employee health status	Healthy climate
Intercept	3.368***	1.944***	3.552***
Control variables			
Employee gender	−0.015 (0.021)	0.079 (0.058)	0.022 (0.015)
Employee age	0.031 (0.018)	0.068 (0.051)	0.016 (0.016)
Employee organizational tenure	0.006 (0.010)	−0.013 (0.029)	−0.010 (0.009)
Main predictors			
Health-promoting leadership	0.146** (0.012)	0.121** (0.033)	0.070** (0.011)
Healthy climate		0.449** (0.131)	
Work unit structure			−0.037 (0.021)
Interaction			
Health-promoting leadership × work unit structure			0.270*** (0.025)
R-Square	0.053	0.069	0.252
F	6.619	5.811	26.056
95%*CI*	(0.028, 0.106)		

*Standard errors (SE) of the coefficients are presented in the parentheses.*

*Results from regression analyses were entered into the online utility developed by Selig and Preacher (2008) (Available at: http://quantpsy.org/medmc/medmc.htm). Monte Carlo resampling method (with 20,000 sample repetitions) was adopted to estimate confidence intervals (CIs) at 95% significance.*

***p < 0.001, **p < 0.01.

**FIGURE 2:**
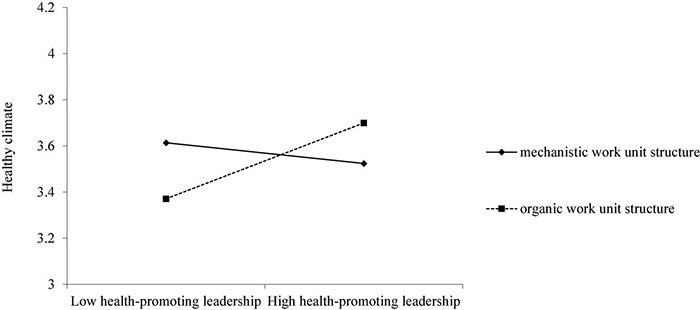
The interaction effect of health-promoting leadership and work unit structure on healthy climate.

Hypothesis 4 theorizes that work unit structure moderates the relationship between health-promoting leadership and healthy climate. As shown in Table 3, the interactive term of health-promoting leadership and work unit structure significantly predicts healthy climate (*b* = 0.270, *SE* = 0.025, *p* < 0.001). Figure 2 illustrates the form this interaction by plotting the simple slope at “high” (i.e., + 1 *SD* from the mean; organic work unit structure) and “low” (i.e., −1 *SD* from the mean; mechanistic work unit structure) values of work unit structure. Health-promoting leadership is positively related to healthy climate when work unit structure is organic (*b* = 0.195, *SE* = 0.018, *p* < 0.001); whereas health-promoting leadership is not significantly related to healthy climate when work unit structure is mechanistic (*b* = −0.05, *SE* = 0.014, *p* < 0.05). Therefore, Hypothesis 4 was supported (Figure 2).

## Discussion

### Theoretical Implications

Given health-promoting leadership is an emerging research field in recent years, there is much theoretical knowledge need to be supplemented. The theoretical contributions made by this study are threefold. First, unlike previous studies, this study investigated the positive impact of health-promoting leadership on employee health status from the perspective of social information processing theory. Extant studies usually theorize health-promoting leadership as a series of explicit leader behaviors that promote employees’ health (e.g., individualized care) (Yao et al., 2021), which limits our knowledge about what social information can such leadership deliver to employees (Winkler et al., 2014). In fact, in addition to direct health-related behaviors, health-promoting leadership also engage in indirect health interventions such as developing a healthy work environment (Jiménez et al., 2017b; Yao et al., 2021). Hence, it is necessary to understand health-promoting leadership from a social information processing perspective. In sum, this study provides a new theoretical perspective for exploring the consequences of health-promoting leadership.

Second, we associated health-promoting leadership with employee health status and uncovered its mediating mechanism. To date, most studies on the consequences of health-promoting leadership have not brought enough empirical evidence for academia (e.g., Akerjordet et al., 2018; Bregenzer et al., 2019). This study linked health-promoting leadership to employee health status and empirically examined its significant and positive relationship. Moreover, according to the central tenets of social information processing theory (Salancik and Pfeffer, 1978), we verified healthy climate as a mediator of this relationship. This investigation helps us gain insights into the relationship of health-promoting leadership and employee health. More frankly speaking, our study empirically demonstrates that health-promoting leadership is positively related to employee health and healthy climate mediates this relationship.

Third, we provide a nuanced understanding about when health-promoting leadership has a stronger influence on employee health status. We investigated the boundary condition of health-promoting leadership by focusing on work unit structure (Aryee et al., 2008). Specifically, we illustrate that the relationship between health-promoting leadership and healthy climate will be more salient when the work unit structure is organic. To our best knowledge, little research has discussed the boundary condition of health-promoting leadership. Work unit structure is theoretically rational to be a moderator because it determines whether the work environment is explicit or ambiguous (Chaurasia et al., 2020). Hence, we provide novel and promising knowledge about when health-promoting leadership is more effective.

### Managerial Implications

This study provides several useful practical implications. First, we suggest leaders to develop their health-related philosophy, skill, and behaviors. By doing so, they are more likely to become health-promoting leaders that has a direct and significant impact on employee health status. Accordingly, organizations should encourage leaders to engage in more health-oriented behaviors. In health promotion practices, besides organization-level policy such as stuff physical examination, it is also useful to consider the effect of health-promoting leaders (Jiménez et al., 2017a). In sum, given health-promoting leadership has significant influence on employee health status, we hope that more health-promoting leadership will emerge in management practice.

Second, since health-promoting leadership can also facilitate employee health status *via* creating healthy climate, we suggest the practitioners to recognize the importance of healthy climate. This study demonstrates that one key process underlying the effect of health-promoting leadership is the enhanced healthy climate. Healthy climate represents shared perception of organizational health-related policies, procedures, and practices among employees, which has infectious function between stuff (Kerr et al., 2019). In fact, the role of health-promoting leadership is not just to shape a healthy climate. For example, health-promoting leadership usually implement a flexible work arrangement (Akerjordet et al., 2018), which subsequently promotes employee health (Shifrin and Michel, 2021).

Third, we also find that employees tend to rely more on the social information conveyed by health-promoting leaders when the work environment is uncertain. From this point of view, we suggest employees to follow their leaders on how to behave if the work environment fails to provide them clear guidance. Similarly, we inspire leaders that they need to design an appropriate work unit structure if they want to strengthen the effects of their health-oriented behaviors on employees. To be clear, it is especially effective for leaders to develop a work unit structure. Our findings demonstrate that employees have a stronger willingness to follow their leaders’ behaviors when the work unit structure is organic.

### Limitations and Future Directions

Although the present study is at the forefront of understanding health-promoting leadership, it is subject to several major limitations that could guide the directions of future research. First, our samples were drawn in several Chinese organizations. Research has shown that Chinese employees are facing urgent health issues, such as the spread of “996” (Liu et al., 2021). Hence, studying the consequences of health-promoting leadership is meaningful in Chinese organizations. Nevertheless, the uniqueness of our sample also limits the generalizability of our findings. To this point, we encourage future studies to use different samples, preferably drawing samples from different cultural countries, to examine our theoretical model. Since this study only investigated the effect of health-promoting leadership, we also suggest future research to explore the antecedents of health-promoting leadership (Köppe and Schütz, 2019).

Second, we also encourage scholars to investigate our theoretical model by adopting more rigorous methodological designs. That is, although we conducted a time-lagged study, reverse causality cannot be totally ruled out. For example, it is hard to assert that whether the health-promoting leadership promotes employee health status, or whether the high level of employee health status causes supervisors to pay more attention to the health issues. Therefore, future research should adopt longitudinal research design or experiment studies to address this issue.

Third, we explored the mediating mechanism of health-promoting leadership and employee health status from social information processing theory. Although this is a creative attempt, we cannot simply judge that healthy climate is the only mediating mechanism. In other words, there are some other potential mediating mechanisms. We advise scholars to adopt different theoretical perspective to explore the mediating mechanism. The same limitation and suggestion also go to the moderating mechanism.

## Conclusion

This study empirically examines a moderated mediation model that links health-promoting leadership to employee health status *via* healthy climate. We also highlight the moderating role of work unit structure. By investigating the effect of health-promoting leadership from social information processing theory, our findings not only add novel and comprehensive knowledge for research on health-promoting leadership, but also provide useful guidance for health promotion practitioners. We encourage scholars to further investigate relevant topics.

## Data Availability Statement

The raw data supporting the conclusions of this article will be made available by the authors, without undue reservation.

## Ethics Statement

Since we conducted our survey in China, ethical review and approval were not required for the study on human participants in accordance with the local legislation and institutional requirements. However, all procedures performed in studies involving human participants were in accordance with the ethical standards of the institutional and/or national research committee and the 1964 Declaration of Helsinki and its later amendments or comparable ethical standards. We also made sure that all individual participants had included in the study have been informed and signed the informed consent. The patients/participants provided their written informed consent to participate in this study.

## Author Contributions

SL designed and conducted the study and drafted the manuscript. ZS and JX edited the manuscript. PC conducted the data analysis. All authors have read and agreed to the published version of the manuscript.

## Conflict of Interest

The authors declare that the research was conducted in the absence of any commercial or financial relationships that could be construed as a potential conflict of interest.

## Publisher’s Note

All claims expressed in this article are solely those of the authors and do not necessarily represent those of their affiliated organizations, or those of the publisher, the editors and the reviewers. Any product that may be evaluated in this article, or claim that may be made by its manufacturer, is not guaranteed or endorsed by the publisher.

## Appendix

### Items of studied variables

#### Health-promoting leadership (3 items)

1. If I don’t feel well, my supervisor will take immediate action.
2. It is important for my supervisor to avoid health pressure and risks for subordinates.
3. My supervisor reduces the subordinates stress by improving their work (e.g., prioritizing work, avoiding disturbing and planning).

#### Healthy climate (5 items)

1. At my workplace, sometimes we talk with each other about improving our health and preventing disease.
2. Most employees here are very health conscious.
3. Around here they look at how well you take care of your health when they consider you for promotion.
4. My supervisor encourages me to make changes to improve my health.
5. Supervisors always enforce health-related rules (smoking policies, requirements about medical examinations, etc.).

#### Work unit structure (7 items)

1. Open channels of communication with important financial and operating information flowing quite freely throughout the organization.
2. Managers can operate in any way from very formal to very informal.
3. Prefer expert decision-making in certain situations even if it means ignoring line managers for a while.
4. A strong emphasis on getting things done even if it means disregarding formal procedures.
5. Special emphasis is placed on adapting to changes in the environment without consideration of past practices.
6. Control is loose, informal, and strongly dependent on informal relationships and standards of cooperation to get things done.
7. tend to determine the appropriate job behavior according to the needs of the environment and personal characteristics.

#### Employee health status (12 items)

1. Able to concentrate.
2. Loss of sleep over worry.
3. Playing a useful part.
4. Capable of making decisions.
5. Felt constantly under strain.
6. Couldn’t overcome difficulties.
7. Able to enjoy day-to-day activities.
8. Able to face problems.
9. Feeling unhappy and depressed.
10. Losing confidence.
11. Thinking of self as worthless.
12. Feeling reasonably happy.
